# Supplementary material for: The Association of Mobile Health Applications with Self-Management Behaviors among Adults with Chronic Conditions in the United States
Source: Int J Environ Res Public Health. 2021 Sep 30;18(19):10351. doi: 10.3390/ijerph181910351 (PMC8507726; doi:10.3390/ijerph181910351)
Supplement: Supplementary file 1 [file ijerph-18-10351-s001.zip › ijerph-1340541-supplementary.pdf]

Supplementary Materials: Secondary data analysis on the association between the use of the mHealth app and self-management behaviors (action planning and self-tailoring)

One of the example questionnaires of action planning and self-tailoring (e.g., tracking and reaching the goal) from HINTS 5 was "has your tablet or smartphone helped you track progress on a health-related goal, such as quitting smoking, losing weight, or increasing physical activity?" We referred to as self-tracking progress on the health-related goal. This was assessed with the yes/no response to the question. We excluded participants who had inapplicable, missing or error data.

The supplementary table shows 1) the comparisons of self-tracking behavior between individuals with and without the mHealth apps. A higher percentage of those with mHealth apps on their smartphone(s) or tablet(s) reported self-tracking progress on health-related goals (69.3% vs. 15.7%) compared to individuals without mHealth apps (p<0.0001, Appendix Table); 2) After the adjustments for biological and cultural variables (i.e., age, sex, and race/ethnicity), socioeconomic status (income and education), and access to care (health insurance) variables, adults with mHealth apps in their smartphone(s) or tablet(s) were more likely to self-tracking progress on health-related goal when compared to those without mHealth Apps. The adjusted odds ratio (AOR) was 12.13 with a 95% confidence interval (CI) of 7.11-19.08 (p<0.001). Hosmer-Lemeshow goodness-of-fit test showed good model fit (GOF, p=0.9525). These study results are similar to the previous reports. [1–4]

Table S1. Comparisons of Self-tracking Behaviors Between Individuals with and without mHealth Applications

	Individuals with mHealth Apps		Individuals without mHealth Apps		
	n	Wt%	*n*	Wt%	*p* value
Track progress on health-related goal					<.0001
Yes	880	69.3	134	15.7	
No	447	30.7	879	84.3	

Reference List

1. Whitehead,L. & Seaton,P. The Effectiveness of Self-Management Mobile Phone and Tablet Apps in Long-term Condition Management: A Systematic Review. *J Med Internet. Res.* **18**, e97 (2016).

2. Zhai,Y. & Yu,W. A Mobile App for Diabetes Management: Impact on Self-Efficacy Among Patients with Type 2 Diabetes at a Community Hospital. *Med Sci Monit.* **26**, e926719 (2020).

3. Mahmood,A., Kedia,S., Wyant,D.K., Ahn,S., & Bhuyan,S.S. Use of mobile health applications for health-promoting behavior among individuals with chronic medical conditions. *Digit. Health* **5**, 2055207619882181 (2019).

4. MacPherson,M.M., Merry,K.J., Locke,S.R., & Jung,M.E. Effects of Mobile Health Prompts on Self-Monitoring and Exercise Behaviors Following a Diabetes Prevention Program: Secondary Analysis From a Randomized Controlled Trial. *JMIR. Mhealth. Uhealth.* **7**, e12956 (2019).
